# Intersection of Data Science and Smart Destinations: A Systematic Review

**DOI:** 10.3389/fpsyg.2021.712610

**Published:** 2021-07-29

**Authors:** Alexander Aguirre Montero, José Antonio López-Sánchez

**Affiliations:** ^1^Intelligence Tourism and Data Analytics at INDESS (Instituto Universitario de Investigación Para el Desarrollo Social Sostenible) University of Cádiz, Jerez de la Frontera, Spain; ^2^Department of History, Geography and Philosophy at University of Cádiz, Jerez de la Frontera, Spain

**Keywords:** data science, smart destinations, bibliometric review, conceptual analysis, COVID-19 pandemic, data science technologies, marketing data science

## Abstract

This systematic review adopts a formal and structured approach to review the intersection of data science and smart tourism destinations in terms of components found in previous research. The study period corresponds to 1995–2021 focusing the analysis mainly on the last years (2015–2021), identifying and characterizing the current trends on this research topic. The review comprises documentary research based on bibliometric and conceptual analysis, using the VOSviewer and SciMAT software to analyze articles from the Web of Science database. There is growing interest in this research topic, with more than 300 articles published annually. Data science technologies on which current smart destinations research is based include big data, smart data, data analytics, social media, cloud computing, the internet of things (IoT), smart card data, geographic information system (GIS) technologies, open data, artificial intelligence, and machine learning. Critical research areas for data science techniques and technologies in smart destinations are public tourism marketing, mobility-accessibility, and sustainability. Data analysis techniques and technologies face unprecedented challenges and opportunities post-coronavirus disease-2019 (COVID-19) to build on the huge amount of data and a new tourism model that is more sustainable, smarter, and safer than those previously implemented.

## Introduction

We currently travel with many digital devices and media that form part of our daily activity: tourism in the twenty-first century is characterized by a hyper-connected tourist profile. Tourists are heavy users of social networks, producing a huge amount of data, an internet trail, or a digital footprint (Encalada et al., 2017) that companies frequently use to obtain valuable information. This is particularly true in the tourism sector.

This scientific literature review provides an in-depth examination of the intersection between data science and smart destinations, focusing on previous bibliometric studies and systematic literature reviews on tourism and smart destinations (Haobin Ye et al., 2020; Mehraliyev et al., 2020; Bastidas-Manzano et al., 2021; Lee et al., 2021). It also includes references to the evolution of information and communication technology (ICT) and tourism (Buhalis and Law, 2008; Law et al., 2014).

In this review, we aimed to summarize the main features of the cross-analysis of the existing literature on data science and smart destinations, comprehensively answering the following research questions:

Q1: What is the importance in the current literature of the term data science in terms of tourism destination management in line with the concept of smart destinations?
Q2: What are the most relevant keywords, authors, publications, and sources related to this research topic during the last years?
Q3: What are the smart destination management areas with the most significant growth potential in the scientific field for data science techniques and technologies?

These concepts have recently become an area of research focus, which motivated the authors to conduct a systematic literature review based on the application of bibliometric analysis; we believe this technique to be better suited to this study than a meta-analysis (Borenstein et al., 2009). Bibliometric analyses enable retrospective analysis of the research field structure, evolution, and existing academic activity trends (Rousseau, 2001).

There are two different bibliometric forms of analysis: performance analysis and scientific mapping. On the one hand, performance analysis focuses on measuring scientific impact and citations through different indexes. On the other hand, scientific mapping enables the representation of scientific research and its evolution in the intellectual, conceptual, and social fields.

The general objective is to identify the main research trends related to the intersection of data science and smart destinations, which will be achieved by addressing the following specific objectives:

- To analyze the existing bibliography on this subject using clusters by keywords, authors, and countries.
- To indicate emerging research themes on the topic of data science and smart destinations.
- To provide a conceptual map of the intersection of data science and smart destinations, identifying those areas in the scientific field with the most significant potential growth.

To achieve these objectives, we conducted a systematic review on the intersection between data science and smart destinations through bibliometric and conceptual analysis, compiling information from the Web of Science (WoS) using the software VOSviewer (Jan van Eck and Waltman, 2019) and SciMAT (Cobo et al., 2012).

## Materials and Methods

This systematic review study used the WoS Core Collection database and was conducted from October 2020 to January 2021 (FECYT, 2001). This database was selected because, alongside Scopus, it is one of the most extensive databases containing scientific publications with the most significant impact in this research area.

A search for the most appropriate keywords was performed using the Google Keyword Planner and Google Trends tools, as well as searches on specialized blogs such as “Kdnuggets” (Piatetsky-Shapiro, 2021) or “Toward data science” (Mishra, 2018). Although previous bibliometric studies have been conducted related to data science and smart tourism, no studies have jointly analyzed the terms data science and smart destinations from a bibliographic analysis perspective. It should also be noted that, because of the small number of existing studies, when we limited the search exclusively to the two terms “data science^*^” AND “smart destinations^*^,” we were forced to broaden the search using other terms related to information analysis techniques or technologies associated with the term data science. This search criterion significantly enriched the search results, producing a significant increase in the number of articles under study from the 16 direct results of the first search covering 2,028 articles 1995–2021, focusing the analysis mainly on the last 5 years.

**Figure 1:**
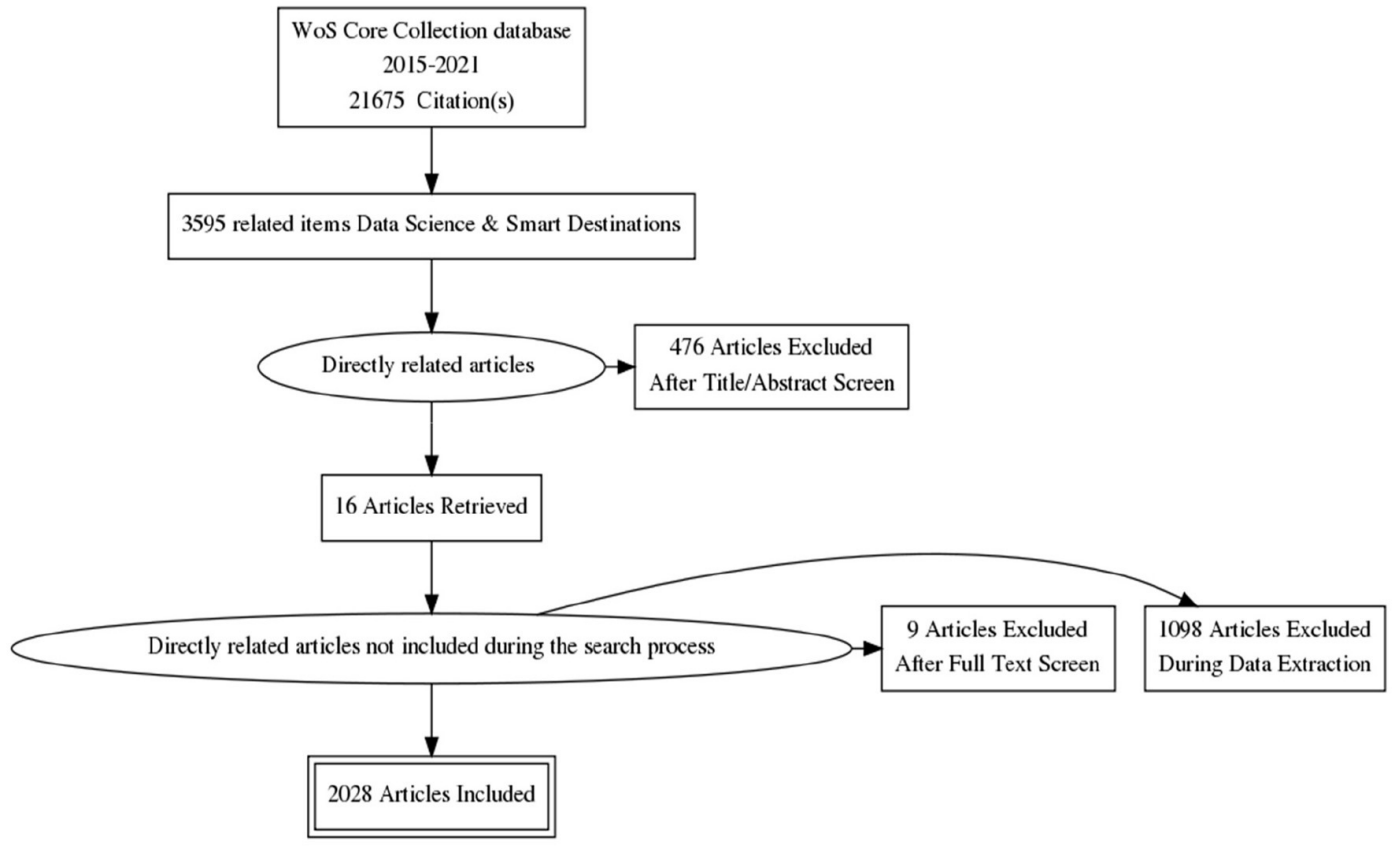
Systematic review flowchart. Source: prepared by the authors using PRISMA Flow Diagram Generator.

Once the most representative terms of data science had been selected, and we carried out a systematic search in WoS using the basic search option, as shown in Figure 1, following the search criteria: TITLE-AND-KEY (“subject” AND “area”). Thus, each query combined a topic and an area, according to the following roadmap:

Topics (35): “data science^*^” + information analysis techniques or technologies associated with the term data science: big data, smart data, small data, business intelligence, cloud computing, data analytics, data exploration, data management, data visualization, data mining, predictive analytics, predictive modeling, sentiment analytics, clustering, multivariate calculus, linear algebra, machine learning, artificial intelligence (IA), blockchain, deep learning, MATLAB, Python, R programming, SAS, SPSS, Statistics, internet of things (IoT), SQL, Qlik sense, tableau, Google cloud computing (GCP), Amazon web services (AWS), Microsoft (AZURE), Hadoop y Docker.
Areas (3): “Smart Destinations^*^”; “Destinations Management^*^”, and “Destination Management Organizations”^*^.
Total (35 topics × 3 areas = 105 total results)

We selected articles and reviews in which these terms appeared in the title, abstract, or keywords. Through searching for the topics mentioned above, we obtained 3,595 publications and manually screened them to select the 2,028 articles directly related to covering the study object 1995–2021.

Once the data were cleaned and believing that we had a representative sample, we continued to work with VOSviewer. This is one of the best current tools for processing keywords and making flow and correlation maps (Hoppen and Vanz, 2016).

Bibliometric analysis was carried out on the sample, using WoS to produce an index comprising the journals that had published articles on “Data Science” and its applications in one of the three search areas: “Smart Destinations,” “Destinations Management,” or “Destination Management Organizations.” The search criteria used guaranteed the inclusion of destination management and destination management organization as research areas, ensuring that the articles selected had an intensive use of data science analysis techniques and technologies.

We aimed to gain an understanding of the evolution of these concepts through the analysis of their leading bibliometric indicators. In addition, we analyzed productivity and impact indicators, showing the number of articles, authors, and citations of prominent journals (Aparicio et al., 2019) to obtain an index of citations related to these topics.

Moreover, we conducted a conceptual analysis that enabled us to study the main concepts or themes related to data science and its applications in smart destinations; this allowed us to investigate the structure, evolution, and trends related to these concepts. For this analysis, we used the SciMAT software. On the one hand, this provided a visual map of the evolution of the concepts or nodes, which encompassed the keywords obtained from the publications of the authors. On the other hand, it enabled us to carry out different strategic analyses of the most relevant concepts, locating them in four categories according to Callon's centrality and density indicators (López-Robles et al., 2019).

Before proceeding with the second part of this research, we needed to conduct two standardization tasks. The first one, related to a new data search in the WoS, involved exporting the search results to SciMAT. We used the advanced search option with the search criteria TS-AND-WC (“subject“ AND “WoS category“). TS = (“data science“ or “big data” or “smart data” or “small data” or “business intelligence” or “marketing data analytics” or “smart destinations” or “destination management” or “destinations management organizations”) and WC= (“Business” or “Economics” or “Hospitality, Leisure, Sport & Tourism”). Among the existing options, these were the most homogeneous criteria related to the first search in the first part of this research. The data obtained on this occasion were journal title, publication date, author details (name and affiliation), article title, keywords, abstract, and citation count.

In the second standardization task, it was also necessary to homogenize the database once exported to SciMAT from WoS to obtain consistent results. Using SciMAT and the Group Set module, we searched for duplicates of the terms by comparing words alluding to the same authors, publications, or sources. It was also necessary to carry out manual work to include keywords of the authors by concepts or specific nodes. In this way, we managed to limit the automatic assignment of many of these terms only by the syntax criteria; although this is very practical, there are occasions where keywords are related to nodes incorrectly (Garrigos-Simon et al., 2018). This analysis provided us with three representations: strategic diagrams, cluster networks, and evolution areas (Gutiérrez-Salcedo et al., 2018).

## Results

Based on the 2,028 articles obtained from the systematic information search published between the years 1995 and 2021, we obtained results that are presented in the following six sections: The first results section introduces the most representative keywords obtained through a search with the 35 themes or techniques of analysis and technologies of data science. The second section relates to publications, while the third section identifies the most relevant authors. The fourth and fifth sections examine the sources, journals, and institutions and the countries that have made the most outstanding contribution to research in this field of knowledge, respectively. Finally, we conducted a conceptual analysis to obtain a global vision of the bibliometric analysis.

### Bibliometric Analysis: Keywords

**Figure 2:**
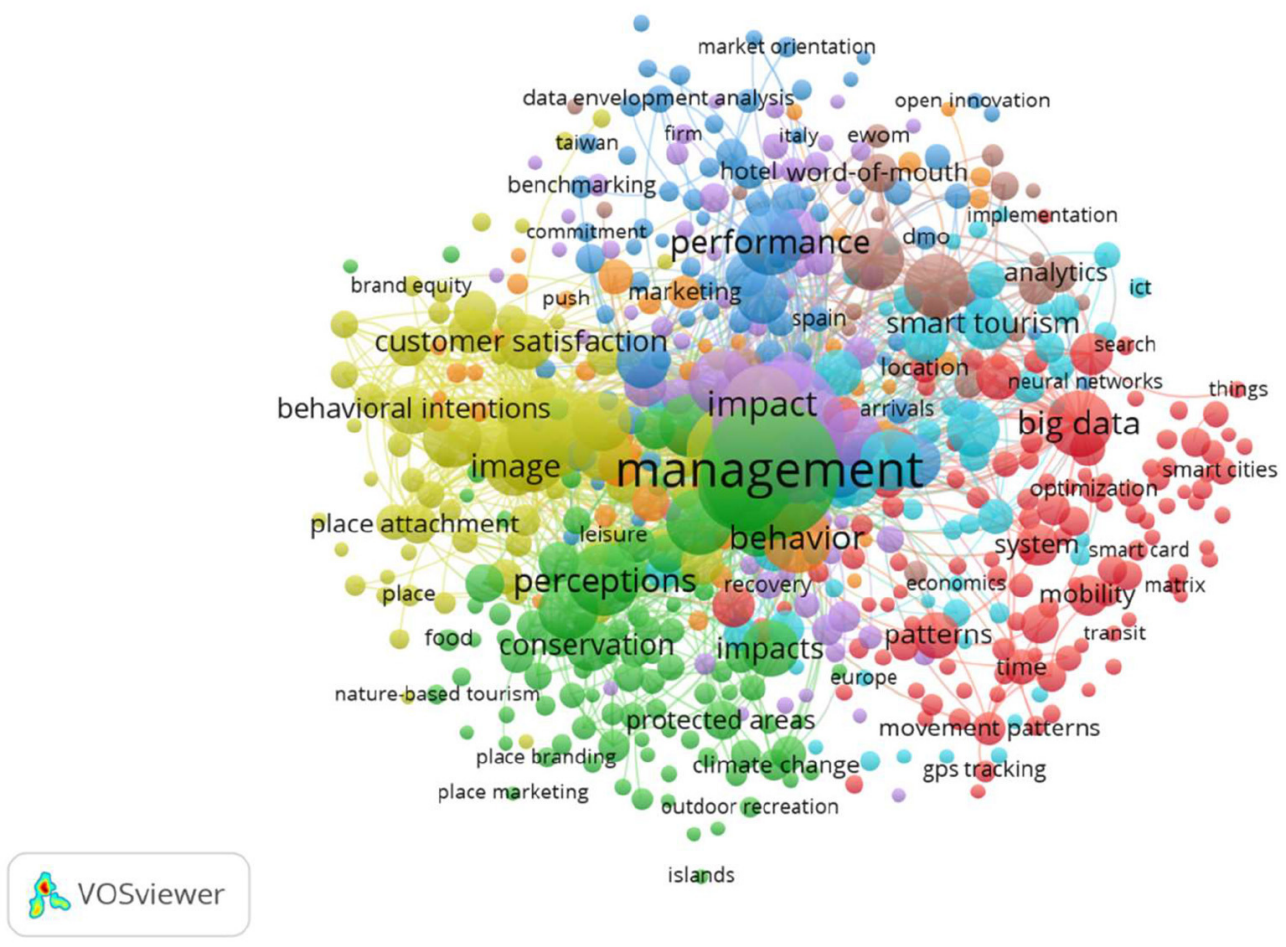
Search keywords analysis (2015–2021). Source: prepared by the authors using VOSviewer, based on WoS data.

The essential parts of this bibliometric study were an intensive search process for the concept of “data science” and the analysis techniques and technologies applied to smart destinations. We obtained the keyword map illustrated in Figure 2 as a result of this thorough search process of the WoS.

We also eliminated keyword duplications in our database using thesaurus files (Salas-Olmedo et al., 2018) to develop a keyword co-occurrence network. Keywords include author keywords (keywords that appear below the abstract) and ISI KeyWordsPlus (words or phrases that frequently appear in the titles of the references of the article but do not appear in the article title itself).

Figure 2 consists of a map divided into five nodes or clusters linking the search keywords. These terms frequently appear in the titles or abstracts of articles or references of the articles related to data science and smart destinations. The lack of centrality of the data science theme on the map is mainly due to this node being rather application-oriented. Another relevant factor is that we were examining the intersection of data science and the concept of smart destinations from a very management-oriented perspective.

Concerning the contents of the different nodes, we can observe the following:

- In the center of the keyword map in green, Cluster 1 is the first node around the term management and related concepts such as sustainability, tourism sustainability, impact, or protected areas. We see a growing interest in the concept of sustainability management as a current lever for the transformation of the tourism sector toward a more sustainable model. It is striking that the concept of climate change appears in this central node related to management, which corroborates the importance that this topic is acquiring.
- Cluster 2 appears in the bottom right in red, where terms related to data science, big data, patterns, and mobility and other terms related to smart cities head the words with the highest relative weight in this node. The term security appears here possibly because of the impact that COVID-19 has had on the tourism industry.
- Cluster 3 in yellow represents the term satisfaction in a broad sense, relating it to other related concepts, such as tourist experience, quality, tourist loyalty, and destination image.
- Finally, there is an agglomeration of terms corresponding to Clusters 4 – blue, Cluster 5—light blue, and Cluster 6—brown, characterized by the keywords performance, smart tourism, and social media. The most recent or trending terms, which are <2 years old, are related to the smart tourism concept and other words related to associated technologies.

We also conducted a temporal comparison between the technologies that form part of the current keyword map of the authors and those that were the keywords in the previous ICT and tourism studies reviews (Buhalis and Law, 2008; Law et al., 2014).

In previous studies, the most prominent technologies were interoperability and ontology technologies, multimedia, mobile and wireless technologies, wireless local area networks (WLANs), functionality and usability, and accessible web technologies. There was the maxim that ICTs can assist in the improvement of service quality and contribute to higher guest/traveler satisfaction. In previous studies, the research focus was on demand–supply, with a clear orientation toward the technologies used by the consumer, referring mainly to those related to the purchase process: need recognition, information search, evaluation of alternatives, purchase decision, and post-purchase behavior.

There was also a focus on other technologies related to companies and destinations, such as eMarketing, e-Strategic management, and web design and analysis.

**Figure 3:**
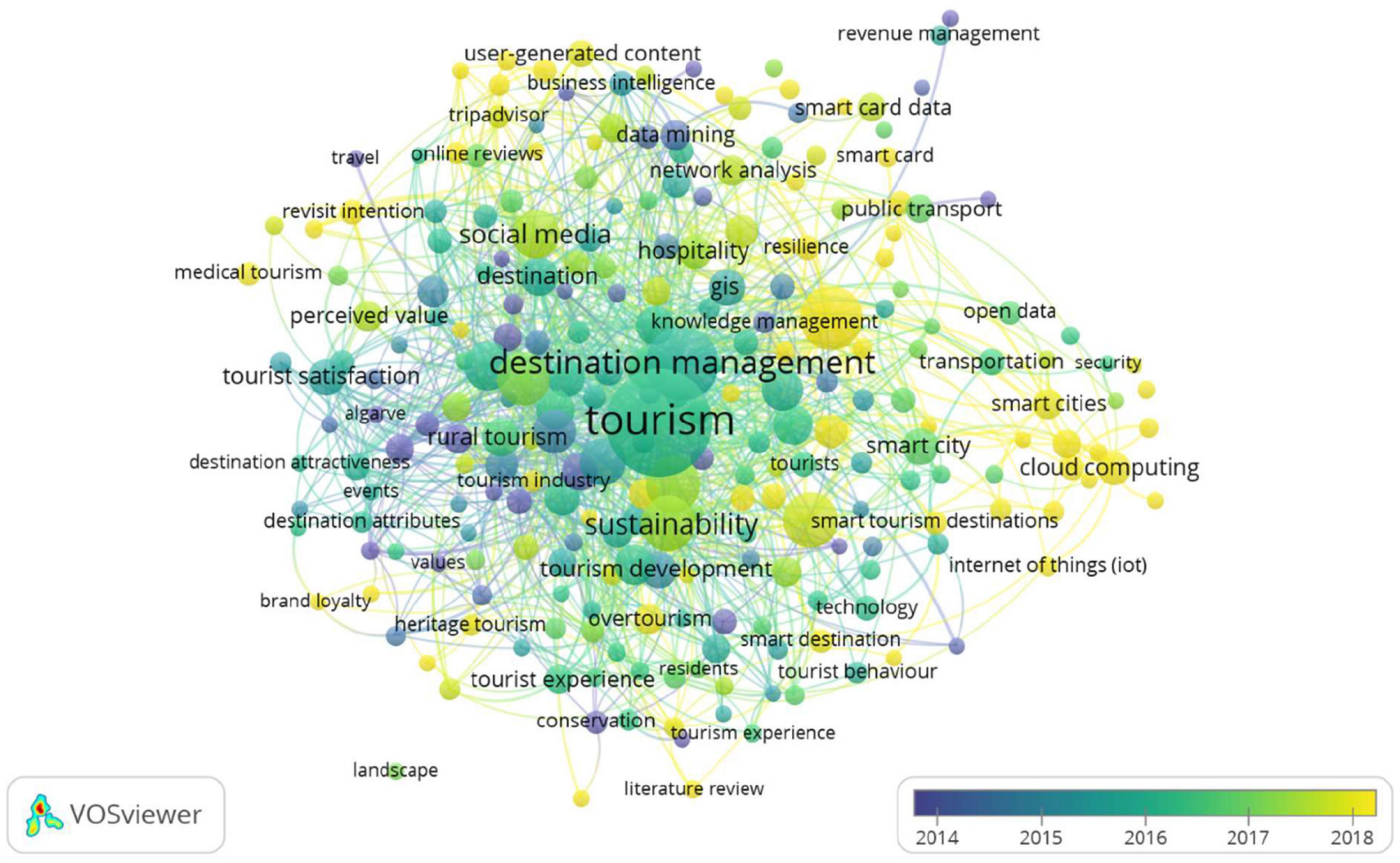
Temporal evolution analysis of author keywords (2015–2021). Source: prepared by the authors using VOSviewer, based on WoS data.

Information and communication technology currently has a holistic vision, comprising an integrated system of network hardware, software, and wearables enabling efficient data processing and communication. Current smart destinations research as shown in Figure 3 is based on the following data science technologies: social media, cloud computing, internet of things (IoT), smart card data, GIS technologies, data mining, business intelligence, open data, artificial intelligence, and machine learning. The fundamental areas where data science techniques are applied in relation to smart destinations are public tourism marketing, mobility-accessibility, and sustainability.

Finally, the importance of the current research on data science for interpreting the huge amount of data generated through social networks has been highlighted. We found numerous related terms in the analysis, among which the most important were online customer journey, tourist experience, online reviews, brand loyalty, perceived value, revisit intention, and user-generated content.

### Bibliometric Analysis: Publication Performance

**Figure 4:**
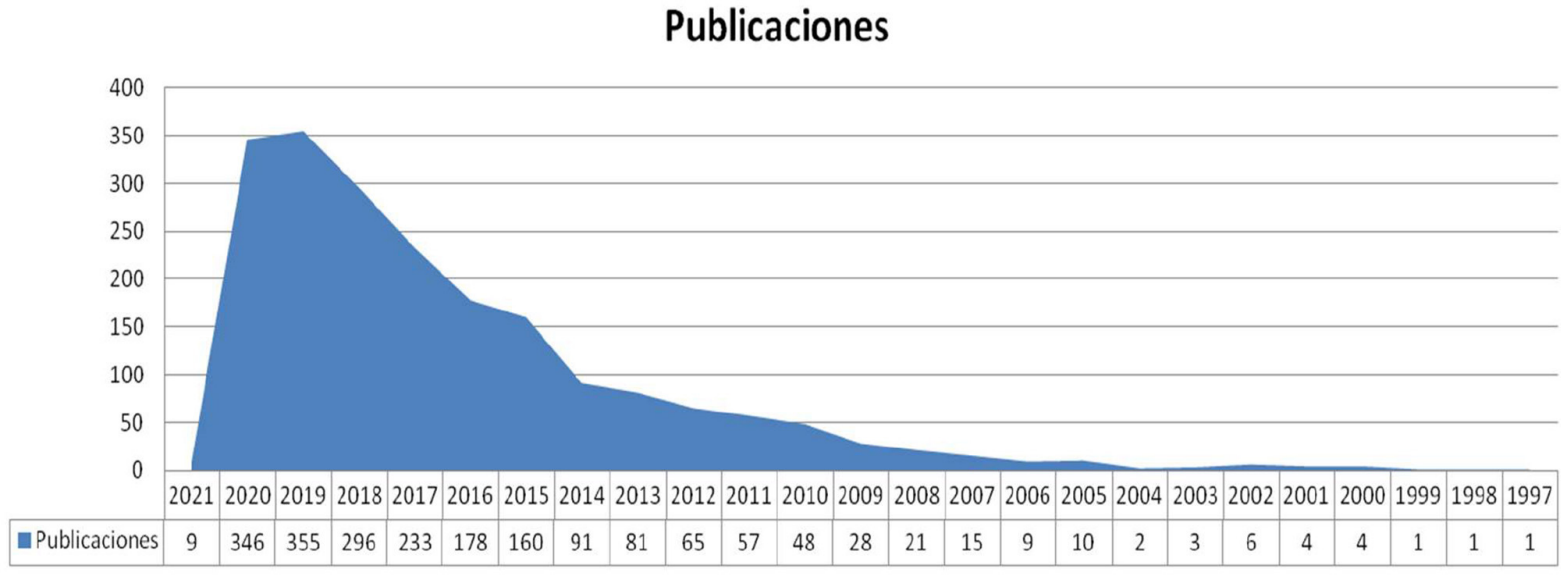
Analysis of publications by temporal distribution (1997–2021). Source: prepared by the authors based on WoS data.

Most of the publications were in the area of knowledge of social sciences and economics. Regarding quantitative component, as shown in Figure 4, the research topic is relatively new. The first publications appeared at the beginning of the twenty-first century. It is possible to identify the first turning point in 2005 when there was a considerable increase in the number of publications. There has been rapid growth since 2015, which indicates a high level of interest in this topic.

**Figure 5:**
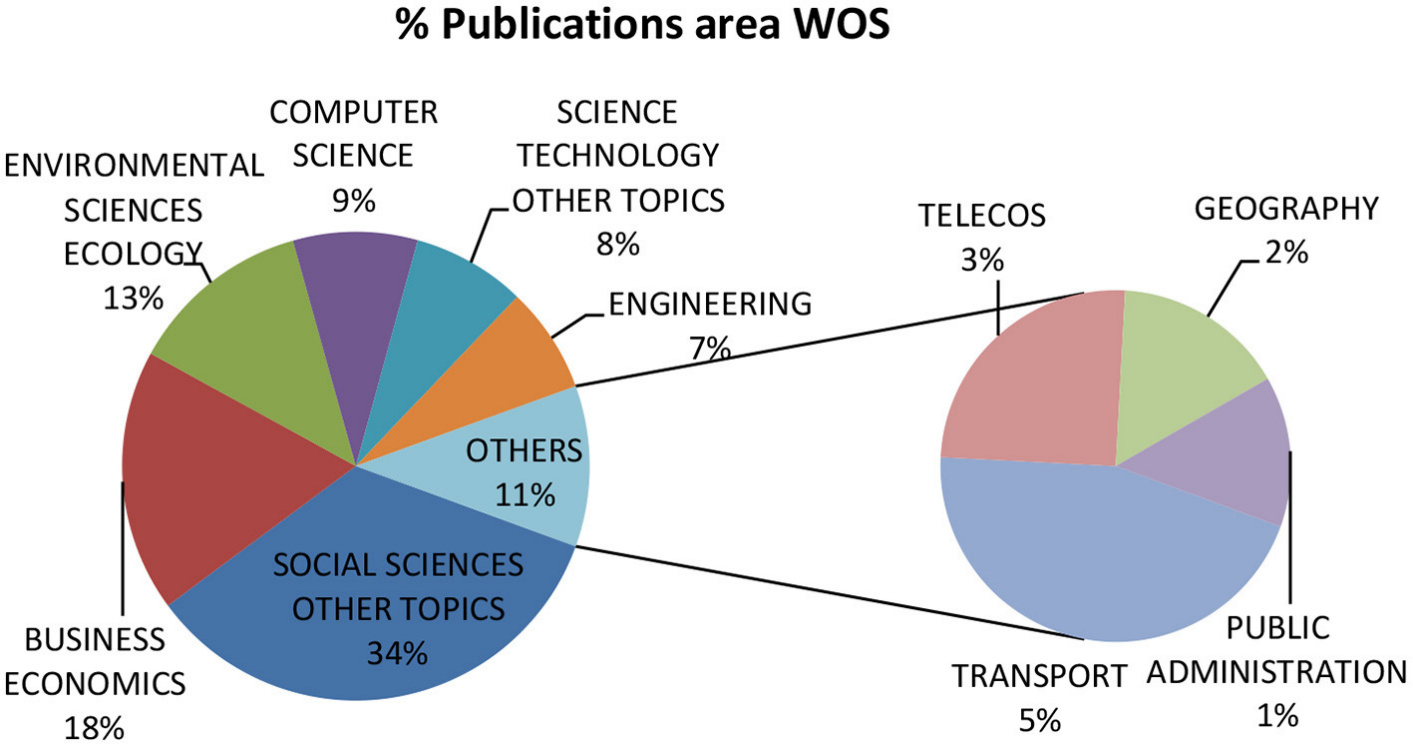
Analysis of publications by WoS research area. Source: prepared by the authors based on WoS data.

Examining the number of articles in the first period (1995–2004) shows that scientific interest in this subject was practically non-existent at this time. In the second period (2005–2014), the number of articles increased from 10 to 91. The third period (2015–2021) corresponds to the last 5 years (including the first month of 2021), where scientific output has reached 355 articles. These publications are classified according to the thematic areas of the WoS database, as shown in Figure 5.

As shown in Figure 5, 34% of publications are in the social sciences, followed by economics (18%) and environmental sciences (13%). This is followed by publications related to technological areas, such as computer science (9%) and other technological topics (8%).

Finally, the total number of citations obtained by the publications in this research area in WoS is 21,675 citations, with an h-index of 63 and an average of 10.69 citations for each item.

### Bibliometric Analysis: Performance Authors

To determine the most productive and relevant authors in terms of publications and citations over the last 5 years, we compiled a list ranking the top 10 authors. The idea was to look for dynamism and trends based on the analysis of the most current data. In this analysis, we found abundant research areas with intensive use of technology.

Table 1 shows the productivity in the last 5 years of authors such as Fuchs Matthias and Lexhagen Maria. Although these authors do not have the highest number of publications, they lead the citation ranking. They are closely followed by other authors who are very active in their number of publications, such as Krask Branislav and Sidor Csaba. For authors whose scientific production is more limited in number but who nevertheless have a high number of citations, we refer to Baggio Rodolfo, Del Vechio Pasquele, and Del Chiappa Giacomo.

**Table 1:** Occurrence of top 10 authors by total link strength (2015–2021).

**Author**	**N^**°**^ of articles**	**Citations**	**Total link strength**
Fuchs, Matthias	7	263	47
Lexhagen, Maria	5	168	45
Krask, Branislav	9	41	33
Sidor, Csaba	9	41	33
Strba, L'ubomir	6	17	26
Baggio, Rodolfo	6	241	22
Del Vechio, Pasquale	6	95	16
Del Chiappa, Giacomo	5	151	12
Marine-Roig, Estela	5	153	9
Law Rob	11	284	8

*Source: prepared by the authors based on WoS data*.

### Bibliometric Analysis: Performance Journals

Table 2 shows the most productive journals concerning the subject studied.

**Table 2:** Top 10 sources for data science/smart destinations (2015–2021).

**Journal**	**N^**°**^ of items**	**% total**
Sustainability	105	5.18
Tourism Management	74	3.65
Current Issues in Tourism	51	2.51
Journal of Destination Marketing Management	51	2.51
Journal of Sustainable Tourism	36	1.77
Tourism Review	35	1.73
Contemporary Hospitality Management	28	1.38
Journal of Travel Research	24	1.18
Tourism Analysis	22	1.08
European Journal of Tourism Research	21	1.04

*Source: prepared by the authors based on WoS data*.

These sources were analyzed according to the number of publications and relative weight of each journal based on the citations obtained. The leading sources are *Tourism Management, Sustainability, Current Issues in Tourism*, and *Destination Marketing Management*, which stand out above the rest of the specialized journals.

### Bibliometric Analysis: Performance Countries

The fifth point of this study focuses on the analysis of the countries that have made the most significant contribution to scientific output in this field of knowledge. From a quantitative point of view, the United States of America is ranked in first place with 310 articles during the period 2015–2021, followed by the People's Republic of China with 283 articles. Following these countries are some of the most touristic European countries, such as Spain (217 articles) and Italy (146 articles).

**Figure 6:**
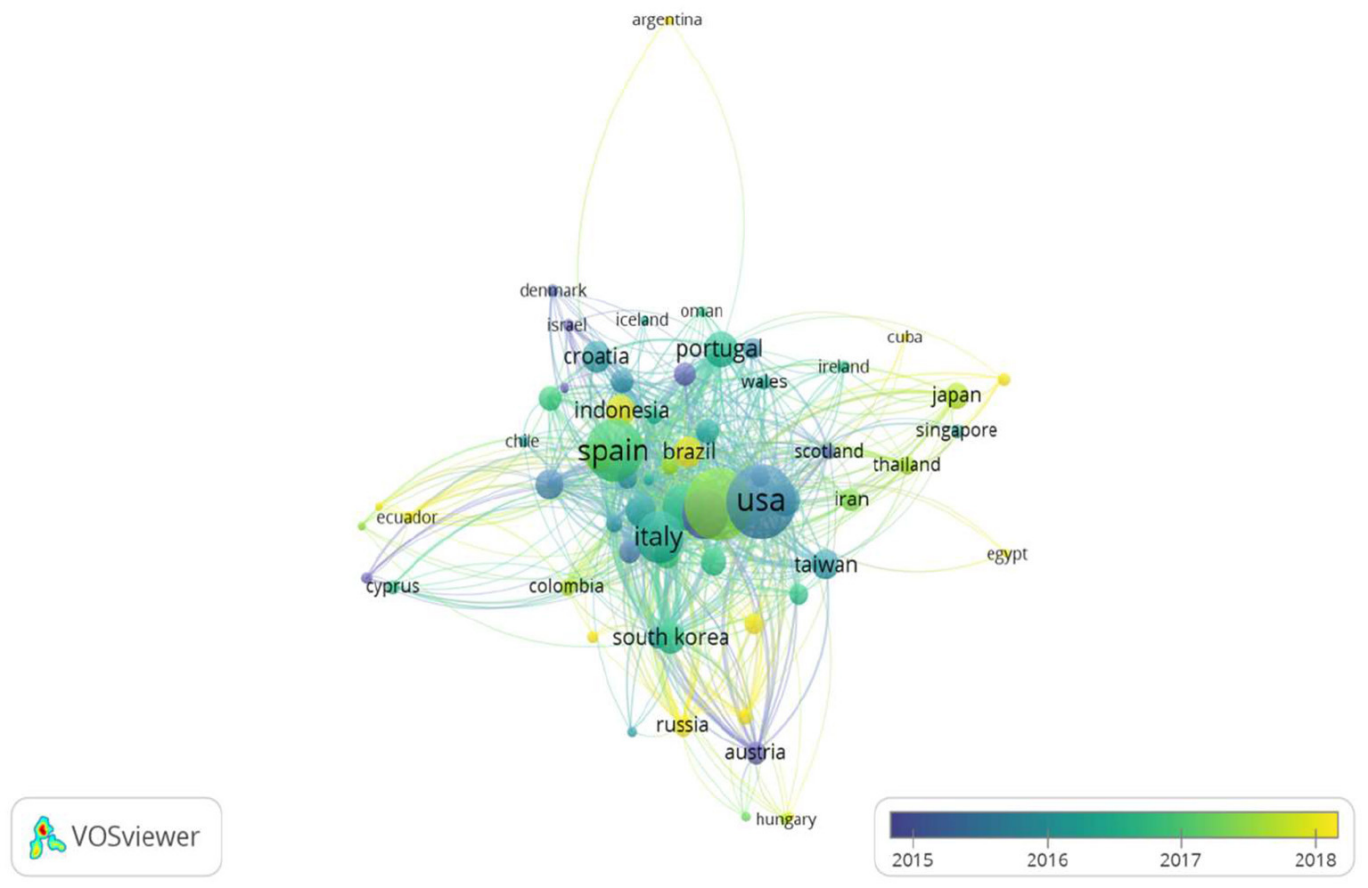
Analysis of country citations by temporal distribution. Source: prepared by the authors using VOSviewer, based on WoS data.

If the quantitative variable analyzes the citations obtained by country, the relative weight of scientific output of each country changes, changing some of the ranking positions, as shown in Figure 6.

In this weighted analysis, Spain is ranked first over the last 5 years with 216 publications and 2,359 citations, 604 points in relative weight publications-citations. The weighted impact of Spanish output concerning the binomial number of articles/citations is higher than that of China and the United States. The main Spanish contribution to this research topic is concentrated precisely in the last 5 years.

### Concept Analysis: Evolution Map and Strategy Diagrams

As a complement to the previous part of the study related to formal aspects, we analyzed the presence, meaning, and relationships of as well as evolution of critical terms recurring in the most cited publications on the intersection of data science and smart destinations. This allowed us to make inferences about the evolution of these concepts over time and to identify the relationships between them and the current trends and driving concepts under which the best research study is concentrated. We also wanted to determine which concepts have the most significant potential for development in the coming years. To do this, we compiled an evaluation map of the terms over time. We also conducted different strategic analyses of the centrality-density of the most relevant concepts.

We have conducted a longitudinal analysis to understand the map of concepts and their evolution during the three stages into which we divided the overall period of study (1995–2021).

The keywords that respond to a global concept were collected into a single group with a unique name. We also used an inclusion index to detect the links between the different concepts (represented by circles) and to define thematic connections (lines).

**Figure 7:**
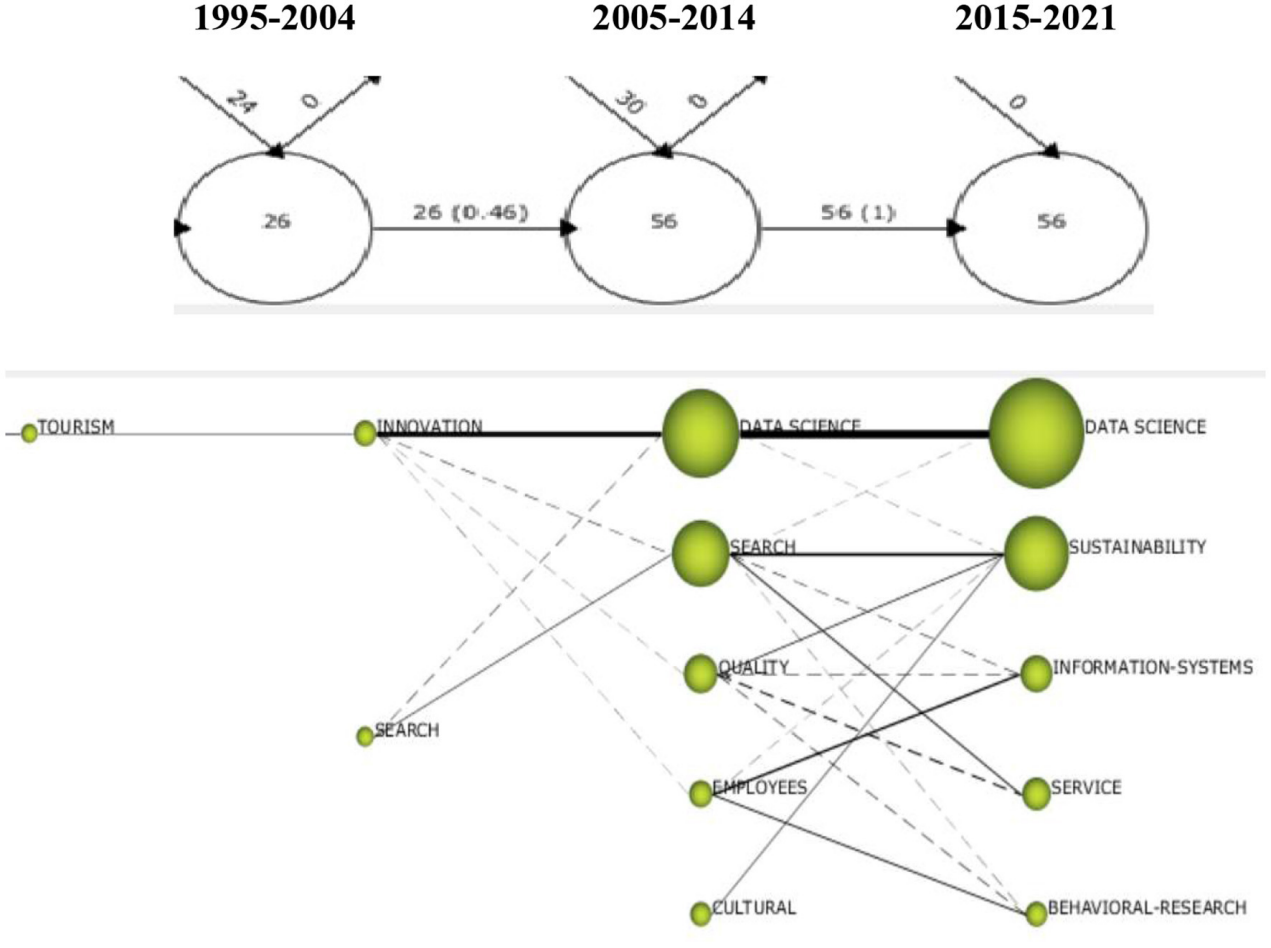
Analysis of thematic evolution (1995–2021). Source: prepared by the authors based on SciMAT.

As shown in Figure 7, the first stage (1995–2004) corresponds to the beginning of research on applications of tourism-related technologies. The related scientific literature focused on the concept of tourism, fundamentally concerning services and satisfaction. From the second period (2005–2014), output in this research area intensified, including 30 new keywords in the analysis, which, added to the 26 maintained from the previous stage, gives 56 terms. These are maintained in the third stage (2015–2021) with data science, sustainability, or behavioral research. It is possible to differentiate concepts with reliable connections to themes from the first period (continuous lines), such as the relationship between tourism, innovation, and data science. However, innovation, quality, or employees had weaker connections (dotted lines), although none of these were the main concepts in the last stage examined to identify the current trends. The size of spheres represents the number of existing articles related to these concepts. There is a clear predominance of the data science concept in the current period, followed by sustainability, tourism information systems, and tourist and visitor behaviors.

**Figure 8:**
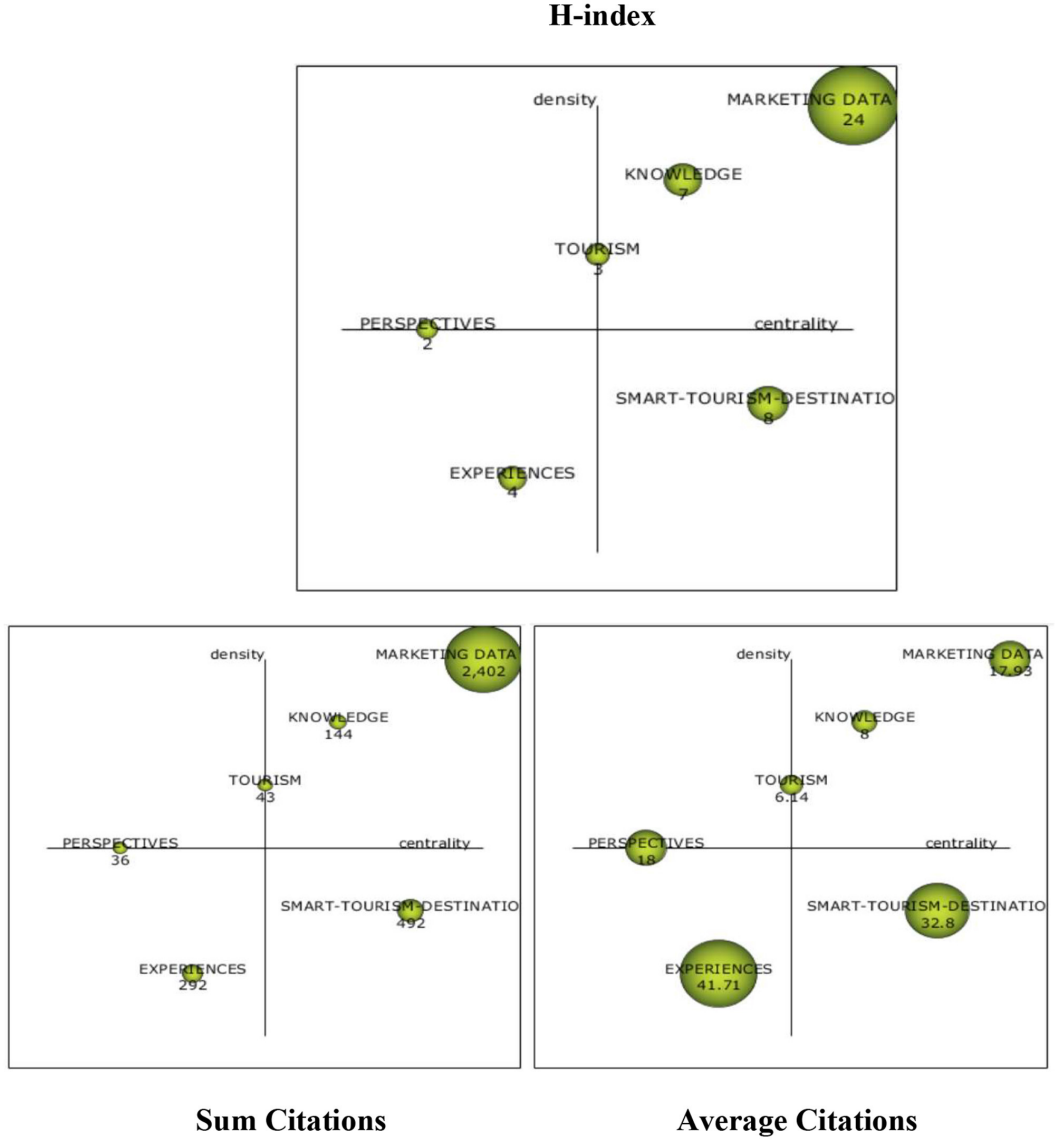
Strategy analysis of keywords by h-index/sum citations/average citations (1995–2021). Source: prepared by the authors based on SciMAT.

As described in the methods section, we conducted a series of strategic density-centrality analyses as shown in Figure 8 of the most relevant concepts during the overall period studied (1995–2021). We studied the density or internal cohesion index of a concept on the ordinate axis through these analyses. On the abscissa axis, we studied the centrality component or external cohesion index. The internal cohesion index of a concept corresponds to internal associations of the concept with the keywords. On the other hand, the external cohesion index is obtained by combining all the equivalence indexes of its external links.

Tourism is the central concept in this analysis and shows both a high internal development and a high iteration rate with the other concepts. In the upper right quadrant, the most dynamic concepts, knowledge and marketing data science, need to expand the feedback process with other clusters or more relevant concepts despite having significant internal development.

In the lower right quadrant, the recurring theme of smart destinations is becoming a driving concept for research. Everything related to experiences in the lower left quadrant is an emerging term in recent years.

**Figure 9:**
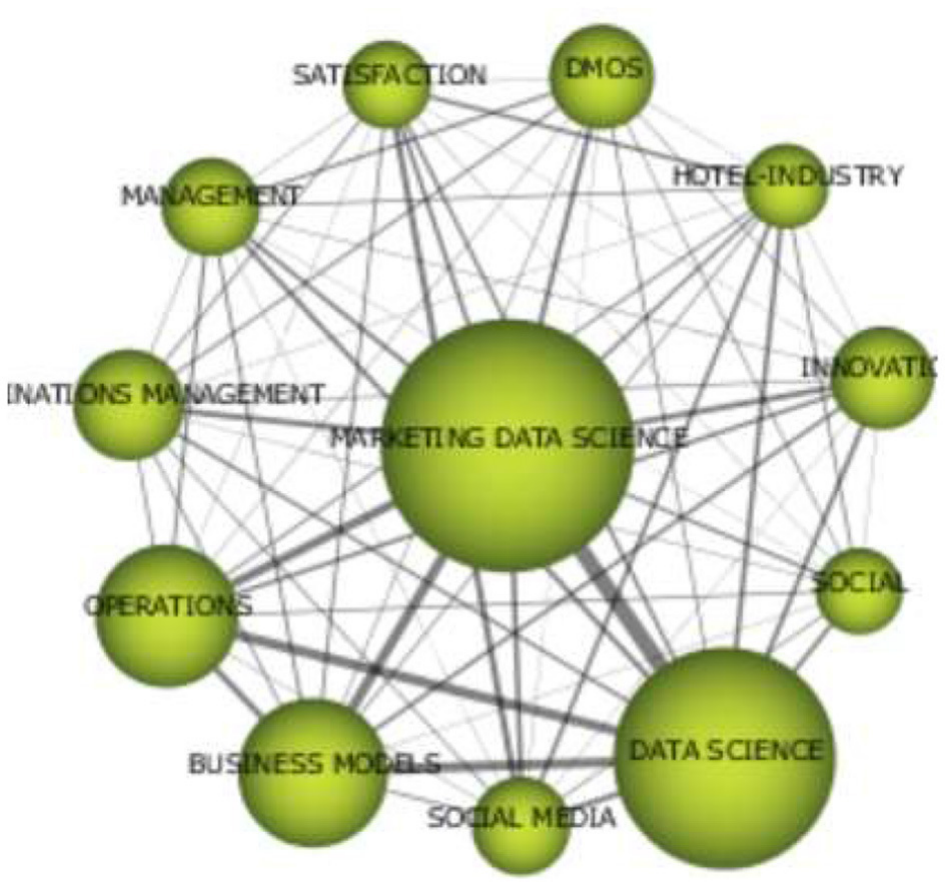
Strategy analysis of publications/thematic evolution (1995–2021). Source: prepared by the authors based on SciMAT.

In the h-index table, marketing data science is prevalent with an h-index of 24, meaning that, out of the total number of publications related to this concept, 24 articles were cited more than 24 times. In terms of the total number of citations, the two previously mentioned concepts of marketing data science and smart tourism destinations stand out again with 2,402 and 492 citations, respectively. A very current concept with an upward trend is tourism experiences, which are materialized in the concept of experiences with 292 citations and the highest relative average score of 41.71. Concluding the conceptualization analysis, Figure 9 presents a network cluster used to identify the most relevant nodes and their interconnections.

A virtuous triangle closely links data science concepts, marketing data science, and operations. This is also strongly linked to the term business models. On the one hand, this information reinforces the data obtained, showing the great potential of data science with other fundamental concepts such as marketing or other operations. On the other hand, data science and business models based precisely on data go hand in hand and are concepts that are becoming increasingly relevant.

## Discussion

Having followed the research process and addressed the relationship between data science and smart destinations, both from a bibliometric perspective and a conceptual analysis perspective, we will now turn to present the main conclusions, answering the research questions set out in the introduction:

- As of 2019, the scientific output related to data science and its applications in smart destinations is more than 300 publications per year. The total number of citations obtained by these publications within WoS is 21,675 citations, with an h-index of 63, averaging 10.69 citations per item.
- Prominent authors in the period 2015–2021 are Fuchs and Lexhagen. Extending the temporal analysis (1995–2021) reveals other established authors such as Gretzel Ulrike and Buhalis Dimitrios.
- The leading sources are *Tourism Management, Sustainability, Current Issues in Tourism*, and *Destination Marketing Management*.
- Over the last 5 years, the most productive country was Spain, with 216 publications and 2,359 citations, followed by the People's Republic of China and the United States.
- The data science technologies on which current smart destinations research are based are social media, cloud computing, IoT, smart card data, GIS technologies, data mining, business intelligence, open data, artificial intelligence, and machine learning.
- Critical areas in research for data science techniques and technologies in smart destinations are public tourism marketing, mobility-accessibility, and sustainability.

In this article, the intersection between data science and smart destinations has been widely analyzed and discussed from different perspectives. The natural evolution of both fields has led to their current interconnection, where data science is viewed as a fundamental lever for the current transformation of the tourism sector. Being aware of this, we have identified the trends in scientific publications in the treatment of both subjects jointly, detecting the main lines of research, together with their evolution and importance in terms of volume and quality of scientific output as proposed in the objectives.

Despite the contributions of this study, some limitations should be mentioned. On the one hand, only publications indexed in WoS have been considered. For future research, we recommend comparing these results with those of other databases, especially Scopus or Google Scholar (Montero-Díaz et al., 2018). Also, SciMAT offers different clustering algorithms and similarity measures selected at the discretion of researchers, as is the case in the clustering of keywords around the fundamental concept nodes, a process carried out based on the criteria of the authors (Cobo et al., 2012). However, to minimize the bias presented, we thoroughly read, reviewed, and analyzed the literature. Finally, the recent dynamism of scientific output on these topics primarily obtained over the last years (2015–2021) prevents the establishment of reliable and definitive conclusions on their thematic evolution. The analysis should be carried out again in the longer term to determine whether the changes and the growth and evolution data identified in this research have been structural, affecting the configuration and evolution of the research field.

## Data Availability Statement

Publicly available datasets were analyzed in this study. This data can be found here: https://www.recursoscientificos.fecyt.es/.

## Author Contributions

AA: management of the database, elaboration of figures and tables using the VOSviewer and SciMAT software, participation in the elaboration and interpretation of the results, and clustering of key terms within the conceptual analysis. JL-S: design and definition of the structure of the research study and research questions and analysis of the results and conclusions. Both authors contributed to the article and approved the submitted version.

## Conflict of Interest

The authors declare that the research was conducted in the absence of any commercial or financial relationships that could be construed as a potential conflict of interest.

## Publisher's Note

All claims expressed in this article are solely those of the authors and do not necessarily represent those of their affiliated organizations, or those of the publisher, the editors and the reviewers. Any product that may be evaluated in this article, or claim that may be made by its manufacturer, is not guaranteed or endorsed by the publisher.
